# Second Harmonic Generation Response in Thermally reconstructed Multiferroic β′- Gd_2_(MoO_4_)_3_ Thin Films

**DOI:** 10.1038/s41598-017-12370-y

**Published:** 2017-09-18

**Authors:** Emerson Coy, Piotr Graczyk, Luis Yate, Karol Załęski, Jacek Gapiński, Piotr Kuświk, Sławomir Mielcarek, Feliks Stobiecki, Bogusław Mróz, Cesar Ferrater, Stefan Jurga

**Affiliations:** 1 NanoBioMedical Centre, Adam Mickiewicz University, Umultowska 85, 61-614 Poznań, Poland; 2 Faculty of Physics, Adam Mickiewicz University, Umultowska 85, 61-614 Poznań, Poland; 3 CIC biomaGUNE, Paseo Miramón 182, 20014 San Sebastián, Spain; 4 Institute of Molecular Physics, Polish Academy of Sciences, M. Smoluchowskiego 17, 60-179 Poznań, Poland; 5 Departament de Física Aplicada, Universitat de Barcelona, Martí i Franquès 1, Barcelona, Spain; 6 Department of Macromolecular Physics, Faculty of Physics, Adam Mickiewicz University, Umultowska 85, 61-614 Poznań, Poland

## Abstract

Gd_2_(MoO_4_)_3_ (GMO) is a well-studied multiferroic material that exhibits full ferroelectric and ferroelastic behavior at room temperature. However, its difficult stabilization in thin films has prevented the study and exploitation of its multiferroic properties in different architectures. Here, we report on the study of GMO thin films deposited on Si(001) substrates by Pulsed Laser Deposition (PLD). The physicochemical properties of the films are discussed and studied. Results obtained by X-ray diffraction, X-ray photoelectron spectroscopy, high resolution transmission microscopy and second harmonic generation show that the orthorhombic (β′-GMO) multiferroic phase can be stabilized and homogenized by post deposition thermal reconstruction. Finally, the reconstruction process takes place via a complex surface mechanism with a clear *leaf-like* behavior.

## Introduction

Modern trends in electronic materials, especially in the field of memories, have focused in the investigation of the physicochemical properties of the so-called multiferroic materials^1–3^, in which the ABO_3_ and A_2_BBO_6_ perovskites have a privilege place^4–9^, dominating most of the research efforts due to the coupling of ferromagnetic^9–15^ and ferroelectric orderings^1,16–22^, which makes them very attractive for information storage devices^23,24^. However, not much attention has been devoted to ferroelectric and ferroelastic coupling and their applicability in non-volatile memory devices or in other electronic applications. One of the main concerns has been the substrate clamping effect, in which stress and ferroelectric polarization is suppressed by the epitaxial strain from the substrate^25^. Nevertheless, several studies have shown ferroelastic switching in thin films^26–28^ showing the potential applicability of these materials in multifunctional heterostructures^29,30^ or artificial multiferroic architectures^31^. Moreover, in materials such as BiFeO_3,_ PbTiO_3_ and BaTiO_3_ the ferroelastic domains are known to play an important role in facilitating the coupling between the polarization and the magnetization via the ferroelastic switching^32,33^, aspect which is highly desirable in non-volatile memories^34,35^. Nevertheless, these traditional ferroelectrics have very limited ferroelastic behavior, mainly due to the need of epitaxial strain in their thin film stabilization, which renders the polarization switching only observable in multi-domain structures, which clearly points out to a clamping substrate of effect with negative implications for electronics^28,36^.

Considering all the above, the epitaxial stabilization of high strain ferroelastics is at the core of novel applications in electronics. Here is where one of the oldest and most well known ferroelectric and ferroelastic material, Gadolinium Molybdate Gd_2_(MoO_4_)_3_ (GMO)^22,37–39^, can be of great use. GMO is a full ferroelectric-ferroelastic material with a complex phase diagram^40^ and effective polarization of P_(001)_: 2 µC/cm^2^ and coercive stress of 1MPa^41^. The complex phase diagram it proses is due to its high oxygen content, a critical aspect of the rare-earth molybdates (Re_2_((MoO_4_)_3_)), which is worsen by the stability of several off stoichiometric phases. Nevertheless, the orthorhombic phase of GMO (β′-GMO), with lattice parameters a = 1.388 nm, b = 1.042 nm and c = 1.07 nm^42^, remains the most studied one. Nowadays, GMO has been extensively used as optical material in electronics^43–46^, however, due to the aforementioned concerns, scarce literature has aim to stabilize GMO in thin films and much less for β′-GMO^45–48^. Sol-gel attempts have proven somehow successful, although the use of high temperature was needed in order to stabilize a fully orthorhombic film (>700 °C), no conclusive proof of the ferroelectricity was provided. This was attributed to short cuts in the film^47^, supported by the fact that cracks are a common problem in sol-gel thin films^49^. Moreover, although sol-gel is regarded as low temperature technique^50,51^, the use of high temperature, without a careful study of the temperature annealing on the crystalline quality of the films, leaves much space for investigation and development.

Despite of the fact that the successful stabilization of β′-GMO could resonate in many fields, and be extrapolated to other molybdates, no other deposition techniques, such as pulsed laser deposition (PLD), have been used. Thin films prepared by PLD are typically homogenous, without cracks or discontinuities and highly tunable, due to the several parameters involved in the deposition process^52^. Moreover, PLD is a technique known to favor the formation of stoichiometric films^52,53^ and it is regularly used in the investigation of multifunctional^54,55^ and epitaxial^9,15,56–58^ thin films and nanomaterials. Reasons for which PLD is an ideal candidate for the investigation of GMO thin films.

Following the current trends in multiferroic materials, which have moved towards the inclusion of multiferroic heterostructures in order to profit from well-known effects such as magnetostriction or electrostriction, in this article we focus on to the Ferroelastic – Ferroelectric properties of GMO/Si(001) thin films deposited by PLD and their *ex-situ* thermal annealing. Here we aim to reconstruct the GMO stoichiometry and investigate the changes of morphology of the samples due to the thermal annealing^59^, which is an important aspect of thin film processing^60^ and reconstruction of surfaces aiming for crystallinity^61^, especially for those with oxygen dependency^62,63^. We will show the complex, but controllable, stabilization dynamics of the GMO films and, moreover, provide proof of the ferroelectric nature of a 40 nm thick film on a silicon substrate. Our results are supported by several physical and chemical characterization methods clearly showing the successful stabilization of the multiferroic β′-GMO phase.

## Experimental Methods

### Thin film deposition and thermal annealing

Thin films of 65 nm (0.13 Å × pulse^−1^) were grown from a polycrystalline single phase GMO target, placed at 5 cm from the Si(001) substrate. The experimental set-up used a Nd:YAG solid state laser with adjustable wavelength working at λ = 355 nm. The base pressure was 5 × 10^−8^ mbar and the working pressure was set to 2 × 10^−4^ mbar of pure oxygen. Post annealing processes were performed on a commercially available furnace working in atmospheric conditions. Samples were placed on a ceramic plate and heated to 600 °C with a heating ramp of 10 °C × min^−1^. Samples were cooled down by thermal inertia in atmospheric conditions.

### Physicochemical characterization

Sample characterization was performed by several methods: Crystalline structure was investigated by Materials Research X-ray Diffractometer in Grazing incident configuration (GI-XRD – PANalytical – X’pert^3^) using the Cu Kα radiation working at 45 kV, 40 mA and an incident angle of ω = 0.4°. High resolution transmission electron microscopy was performed with a HR-TEM – JEOL – ARM 200 F. Composition was investigated by energy-dispersive X-ray spectroscopy (EDX detector mounter in HR-TEM) and X-ray photoelectron spectroscopy (XPS – SPECS – Sage HR 100), working with a non-monochromatic X-ray source (Al K_α_ line of 1486.6 eV energy and 350 W). Morphology of the samples was investigated by atomic force microscopy (AFM– Bruker– ICON) using tapping mode and commercial silicon cantilevers. Nanomechanical Topographic images were collected using a Nanoindenter (TI 950 – Hysitron). Maps were collected using the modulus maps option after calibrating the curvature of a Berkovich tip on a flat fused quartz surface (69.6 GPa). Finally, internal structure and non-centrosymmetric nature of the GMO films were investigated by Second Harmonic Generation measurements (SHG – Zeiss – LSM780 NLO) performed with two-photon excitation (Chameleon 680-1080 nm, 140 fs) optics.

## Results and Discussion

**Figure 1:**
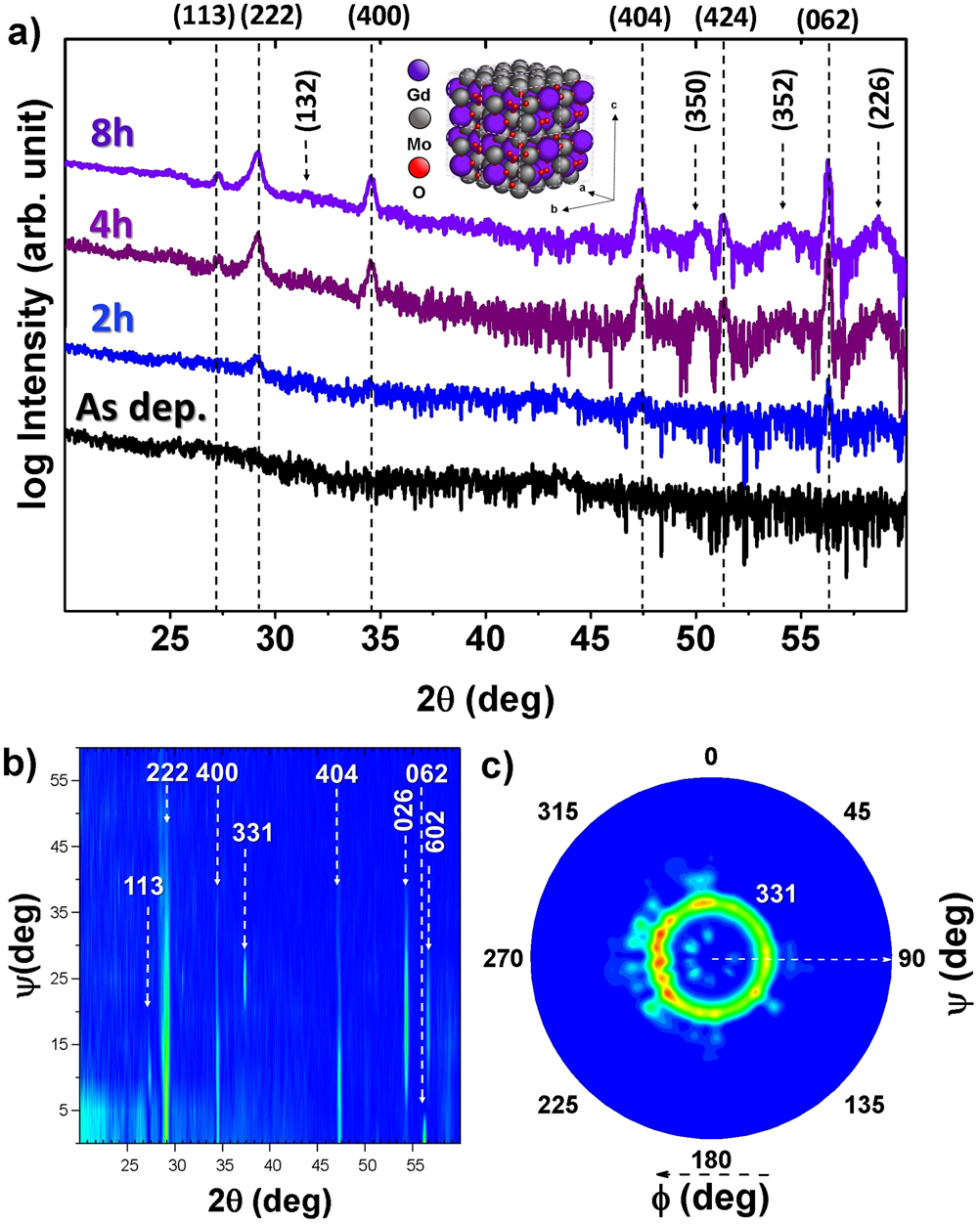
Gi-XRD measurements (**a**) 2thetha scans of samples annealed for different periods of time, peaks are indexed according to nominal β′-GMO position. Inset depicts a 2 × 2 × 2 unit cell of β′-GMO, (**b**) 2θ vs Ψ plots and (**c**) Pole figure of GMO(331) direction performed on the sample annealed for 8 h.

As-deposited samples showed no discernible crystalline features in the available deposition temperature range, RT to 500 °C (Fig. S1), therefore, thin films were post annealed *ex-situ* aiming to study the stabilization of the GMO phases. Samples were collected and post annealed in individual runs for post experiment characterization. Figure 1(a) shows the crystalline changes of the films as a function of the post annealing process by means of GI-XRD. The as-deposited sample shows an amorphous structure. The first crystalline peaks are visible after 2 h of annealing, with the clear appearance of GMO(222) (ICCD: 01-070-1397) and further crystalline directions, such as GMO(400) and GMO(404) which are also observed after 4 h of annealing. The results show a clear polycrystalline film after 8 h of annealing. Although, the crystalline peaks are consistent with those of β′-GMO, several isoforms and off stoichiometric lattices of GMO exist, thus in order to determine identity of the stabilized phase, GI-XRD experiments were performed and are shown in Fig. 1. Figure 1(b) shows the 2θ vs Ψ maps performed on the sample annealed for 8 h, although the sample remain highly polycrystalline in the whole range investigated, a clear peak corresponding to the GMO(331) direction is observed at around Ψ = 22–25 deg. This peak would be observable at that given angle if the out of plane texture of the film was GMO(111). Figure 1(c) examines in detail such reflection by means of polar plots. The ring observed in the graph is a clear signature of high *in-plane* polycrystallinity, with no visible order, but also clearly shows the preferential GMO(111) *out-plane* texture, and moreover, it also suggests the successful stabilization of the ferroelectric β′-GMO phase.

**Figure 2:**
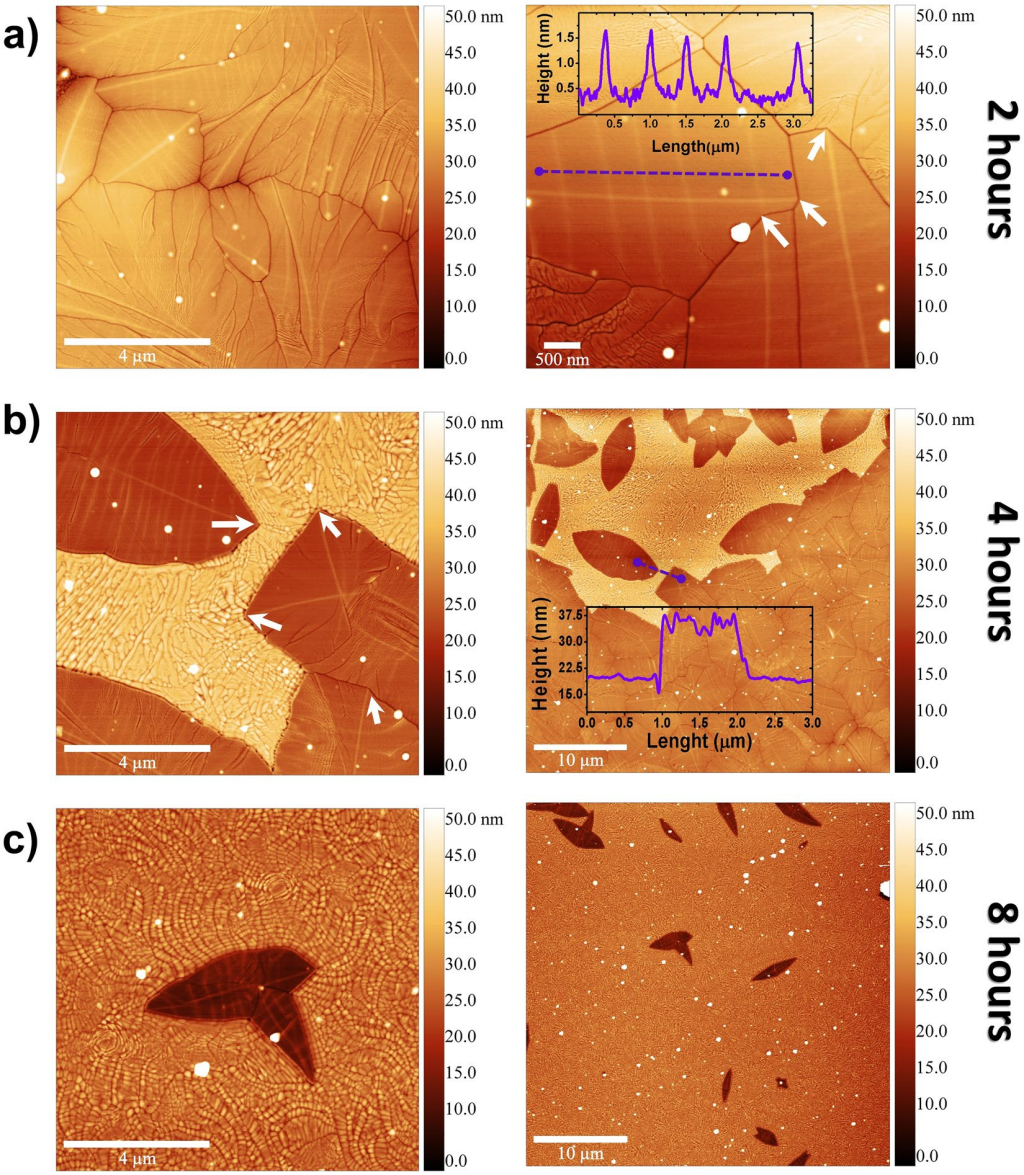
Atomic force micrographs of (**a**) sample annealed for 2 hours, 10 × 10 µm (left) and 5 × µm (right), inset shows topographic profile of the dashed (purple) line, notice the ≈1 nm increment across the profile. (**b**) Sample annealed for 4 hours, 10 × 1  µm (left) and 40 × 40 µm (right), inset shows topographic profile of dashed (purple) line. (**c**) sample annealed for 8 hours, 10 × 10 µm (left) and 40 × 40 µm (right).

The evolution of the films morphology vs annealing time was investigated by AFM showing the clear apparition of a well described secondary phase after 2 h of annealing, Fig. 2(a), while the as deposited surfaces show no distinctive features, Fig. S2. Images show that the surface reconstruction of the samples, by longer periods of thermal annealing, follows specific nucleation points that resemble a *leaf-like* topography. This topography quickly gives rise to a new phase, which slightly protrudes from the film surface. This allows the clear observation of two distinctive populations, one quite flat and with *leaf-like* features, and the second one rough and overpopulated by molds. A clear coverage increment of the rough phase is observed with the longer annealing time, which correlates well with the increment of crystallinity observed in the XRD studies.

The surfaces seem to follow the apparition of *midrib-like* structures, from which *veins-like* are shown to protrude, as clearly observed in the sample annealed for 2 h, Fig. 2(a). It seems however, that *veins* cross individual grains in no preferable path, while on the other hand, a clear tendency is observed for the *midribs*, which seem to interconnect by specific nucleation points. In Fig. 2(a,b), some nucleation points of *midribs* are marked by white arrows, these points seem to appear at turns of the grain boundaries while the *veins* are present at several areas following or interconnecting places with no clear trend. It is important to notice that both, *midribs* and *veins* have ≈1 nm height over the grain surface, Fig. 2(a) inset, and are present in every grain of the studied topography. It is clear though, by observing the samples annealed for 4 and 8 hours, Fig. 2(b,c), that the roughness of the appearing phase increases with longer time of thermal annealing (from R_q_ = 0,5 to 4,4 nm) as well as its height (z = 17 nm). This increment of roughness and height clearly suggests not only crystalline changes, as shown in Fig. 1, but also chemical variations, which seem to be induced, or mediated, by the *midrib* and *veins* structures.

Further research on thinner films (5 nm and 10 nm) showed that the films seem to follow a Pseudo-dendritic growth (Fig. S3), which has been already observed in some oxides^65,66^ and molybdates materials^67,68^. Moreover, these studies allowed to determine the coalescence threshold of the films, with a critical thickness of ≈15 ± 3 nm and the similar dendritic behavior in thinner samples. Additionally, as expected, thickness plays an important role in the reconstruction mechanism, monotonically reducing the annealing time required for crystallization with the decrement of thickness in the films. Moreover, in the reconstruction mechanism, both the melting front of the crystalline phases and the different growth/nucleation energy of each surface play a major role in the reconstruction. In principle, both the crystallization and dendritic growth of the GMO films are taking place across the surface of the samples. This can be appreciated in Fig. 2(a) in which early stages of dendrites and Fig. 2(b) with well-formed ones are observed, as well in the Fig. S3. It is important to notice that the propagation of the dendrites takes place along the boundaries, which seems to be assisted by the higher oxygen content of the reconstructed phase.

**Figure 3:**
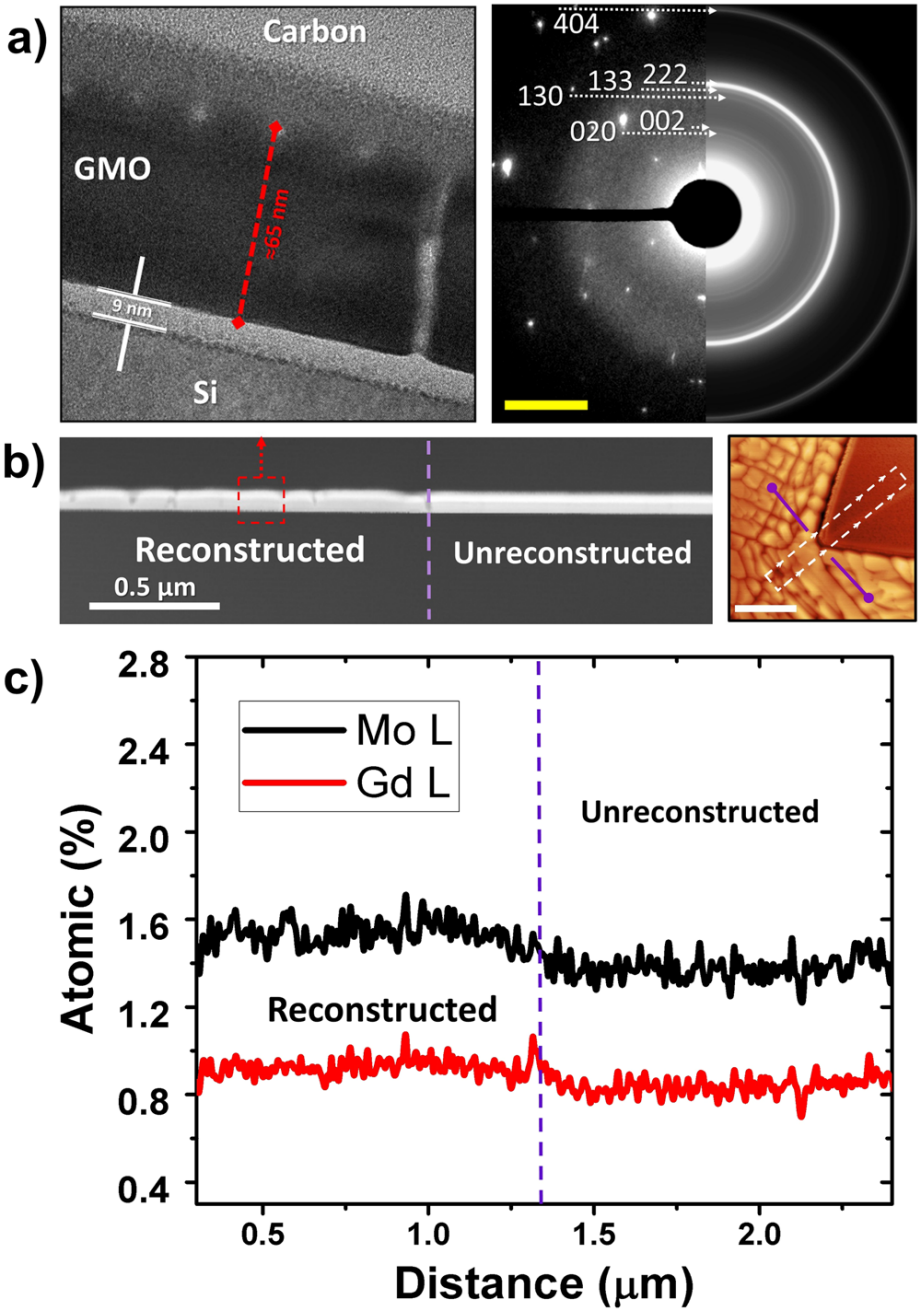
(**a**) HR-TEM image of sample annealed for 8 hours (left) and collected SAED pattern, scale is 2 × nm^−1^ (right), SAED pattern is divided in two sections, as collected (left side) and processed pattern (right side). This was done in order to allow easy identification of the polycrystalline pattern^64^. (**b**) Cross-section Dark field (DF) image of the sample, clearly showing the presence of both reconstructed and unreconstructed areas in the sample (left). Lamella for HR-TEM observation was collected from the dashed region in the AFM image (right) (**c**) Extracted values of EDX compositional mapping for Gd and Mo (normalized to O at.%) in the region shown in DF image (covering reconstructed –left and as deposited –right areas), both with similar scale (500 nm).

HR-TEM experiments were performed on the sample annealed for 8 hours. Figure 3(a,b) show cross section HR-TEM analysis and electron diffraction results. The microstructure remains highly polycrystalline, as shown in the Selected Area Electron Diffraction (SAED) pattern Fig. 3(a), and in the processed pattern (right side). A clear presence of the GMO(222), in the out of plane direction is observed. Additionally, clear boundaries are observed connecting the free surface and the substrate. Notice that the intermediate layer between the carbon top and the film is gallium damage induced to the samples’ surface during the FIB preparation (Fig. S4). Figures 2(c) and 3(b) show the Gd and Mo relative at%, normalized to the total O at.%, along the boundary of the two visible phases in the 8 h sample. This study allows to see stoichiometric changes between the reconstructed and original phases. Notice that the spectra was taken from a straight line covering both phases, large magnification of this area is shown in Fig. 3(b). In Fig. 3(c) it can be clearly seen how the Mo content changes from 1.6 at% to 1.3 at%, while the Gd drops from 0.9 at% to 0.8 at% from phase to phase, the nominal ratio of the ferroelectric β′-GMO is *A*_Gd:Mo:O_ = 2:3:12, thus, although the quantification of oxygen by EDX is not as accurate as for heavy elements^69^, the proportion of unreconstructed and reconstructed phases are Gd_0.9_:Mo_1.6_ = 0.562 and Gd_0.8_:Mo_1.3_ = 0.615 respectively, showing a clear difference in stoichiometry and more importantly, showing that the reconstructed phase, closely resembles the nominal atomic ratio, Gd_2_:Mo_3_ = 0.66, of the multiferroic β′-GMO.

**Figure 4:**
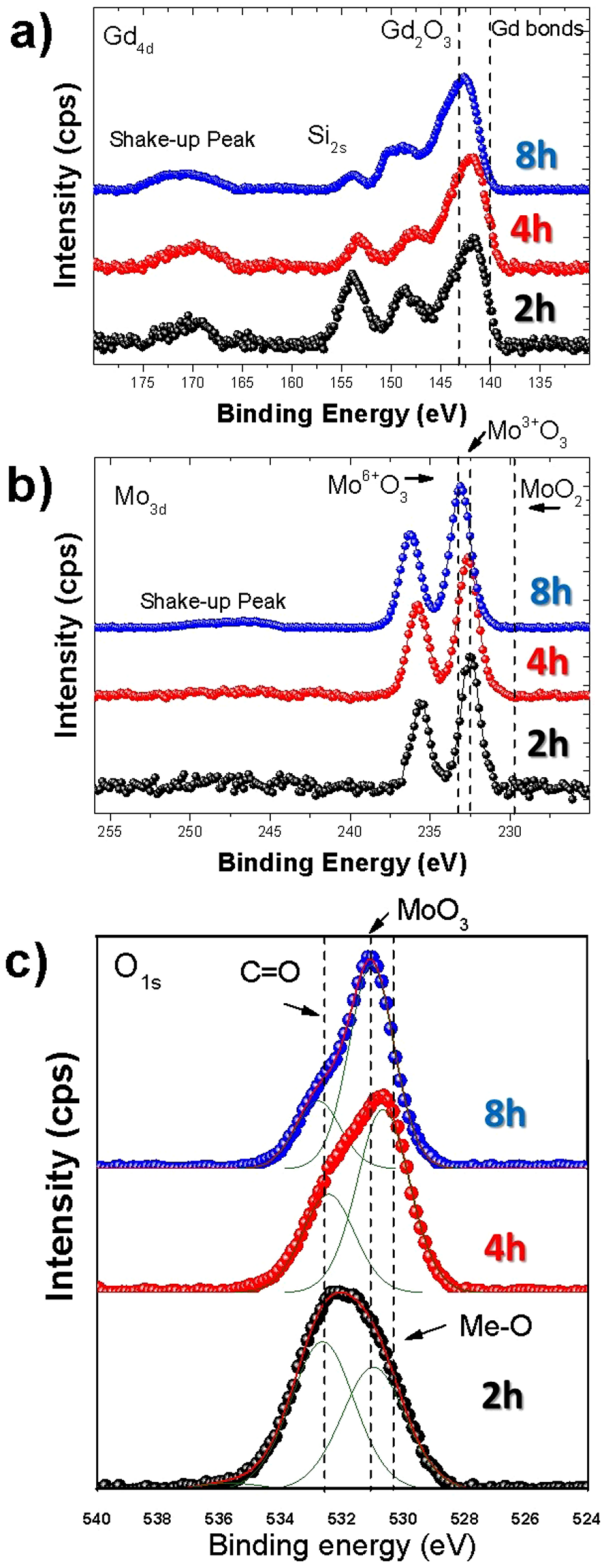
High resolution XPS spectra of (**a**) Gd 4d, (**b**) Mo3d, (**c**) O1s, for samples annealed for 2, 4, 8 hours.

In order to clearly investigate the stoichiometry of the samples, XPS measurements were performed, Fig. 4(a) shows the Gd 4d spectra for the samples annealed at 2, 4 and 8 hours. The spectra cannot be fitted due to the asymmetric shape of the peaks. Nevertheless, the Gd 4d peak position shows a progressive displacement towards the nominal position for the Gd_2_O_3_ species at around 143.3 eV^70^, following the thermal annealing process of the samples. Mo 3d peak, Fig. 4(b), also shows a clear reconstruction due to the thermal annealing, in which Mo^3+^O_3_^40^ peaks move towards a the Mo^6+^O_3_^69^ position. This trend is also visible in the O1s peaks, Fig. 4(c), in which MoO_3_^69^ is clearly visible after 8 hours of annealing. Therefore, it is clear that the thermal annealing is able to reconstruct the stoichiometric ferroelectric β′-GMO phase, Table S1.

**Figure 5:**
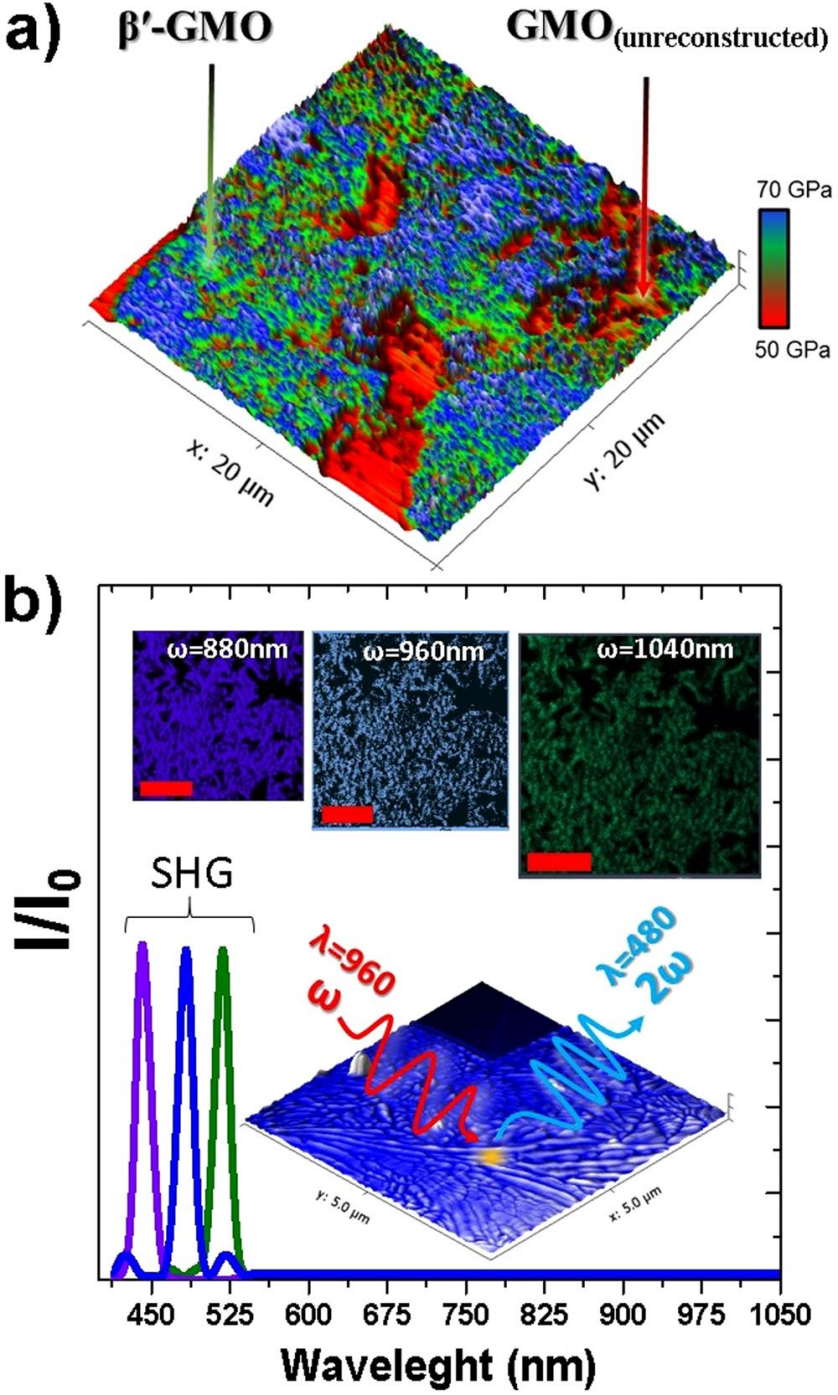
(**a**) Mechanical modulus mappings of β′-GMO sample annealed for 4 hours. (**b**) Normalized intensity (I_0_) of SHG response of the sample annealed for 8 hours, Inset shows the nominal mechanism and topographic areas from where SHG signal was recorded. Scale bar is 10 µm, λ is the incident photon wavelength and ω its associated frequency $(\omega =c\times {\lambda }^{-1})$.

Ferroelastic properties of β′-GMO have been widely studied in the past^30,41,45,46^ giving a rather clear picture of the general mechanical response of the material, with an elastic modulus of ≈62.5 GPa^42^. Nanomechanical topographic maps have been performed on the sample annealed for 4 hours, in order to evaluate the mechanical response of the films. Figure 5(a), shows the modulus mapping results of the measurements, and although the topography is unclear, due to the high radius of the indenter tip, the mechanical response of the reconstructed area (higher topographic regions) clearly corresponds to the nominal β′-GMO elastic modulus. However, due to the polycrystallinity and low dimensionality of the films a strong proof of its ferroelectric response has to be provided.

In the past few years SHG has emerges as an efficient way to study ferroelectric materials and polar-nonpolar oxide interfaces^71^. SHG is a nonlinear optical process in which two photons with a frequency (ω), combine in order to generate a photon with twice the energy and therefore, half the wavelength (2ω) Fig. 5(b). This process is determined by the ${P}_{i}(2\omega ,z)={\varepsilon }_{0}{\chi }_{ijk}(z){E}_{j}(\omega ,z){E}_{k}(\omega ,z)$, where z the direction perpendicular to the interface, ${\rm{P}}(2\omega ,z)$ is the induced second-order polarization response, ${\rm{E}}(\omega ,z)$, is electric field of the incident photon and χ is the susceptibility tensor^71,72^. It is clear then, that this behavior is observed in materials without inversion symmetry, non centrosymmetric, hence, since ferro and piezo electrics require broken inversion symmetry, is an excellent method to probe ferroelectric materials. Due to the highly polycrystalline nature of the β′-GMO phase presented in the samples, and the nominal easy axis of polarization along the GMO(001) direction^39,41,73^, it is sensible to assume that polarization would be aligned randomly on the surface, thus no anisotropy could be expected. Nevertheless, the signal would confine to the β′-GMO phase, while the as deposited material, or the off stoichiometric phase, will not have any ferroelectric response. Figure 4(b), shows the topographic SHG response of the sample annealed for 8 h. The wavelengths used were 880, 960 and 1050 nm, thus, the SHG detection range was set to 440, 480 and 525 nm respectively. Topography images, Fig. 4 inset, clearly show dark regions in which SHG signal is missing. Those regions are the off stoichiometric GMO, while the bright, SHG regions are from the stoichiometric ferroelectric β′-GMO. The collected wavelengths fall within the previously observed range for orthorhombic β′-GMO^74^, clearly showing the distinctive ferroelectric nature of the films.

## Conclusion

In conclusion, we have explored the physicochemical properties of thermally reconstructed ferroelectric β′-Gd_2_(MoO_4_)_3_ samples. We found an unique topographic process leading to a complete reconstruction of the ferroelectric GMO phase, from a fully amorphous, as deposited structure, to a granular ferroelectric one. The reconstruction takes a sort of *leaf-like* behavior, in which *midribs* and *veins* are observed, and assumed to work as nucleation fronts, as well as allow the oxygen diffusion and inclusion on the reconstructed surfaces. Finally, the ferroelectric nature of the films was assessed by SHG, clearly showing their non centrosymmetric nature, which coupled with the crystalline and stoichiometric studies, clearly shows the successful reconstruction of the multiferroic (ferroelectric-ferroelastic) β′-GMO thin films.

Our study shows an important bench mark for this material and the rare earth molybdate family. Additionally to the multiferroic fields, where β′-GMO stands as the only known full ferroelectric and ferroelastic material. Moreover, our results open the possibility for much physics and electronic applications to be explored. Further studies are needs to investigate the high temperature/high oxygen deposition conditions of β′-GMO by PLD and the influence of epitaxial strain in its physicochemical properties.

## Electronic supplementary material

Supplementary information

## References

[1] Scott JF (2013). Room-temperature multiferroic magnetoelectrics. NPG Asia Mater..

[2] Cheong S-W, Mostovoy M (2007). Multiferroics: a magnetic twist for ferroelectricity. Nat. Mater..

[3] Ramesh R, Spaldin NA (2007). Multiferroics: progress and prospects in thin films. Nat. Mater..

[4] Vasala, S. & Karppinen, M. A2B′B″O6 perovskites: A review. *Prog*. *Solid State Chem*. doi:10.1016/j.progsolidstchem.2014.08.001 (2014).

[5] Khomskii DI (2006). Multiferroics: Different ways to combine magnetism and ferroelectricity. J. Magn. Magn. Mater..

[6] Dong S, Yu R, Yunoki S, Liu J-M, Dagotto E (2008). Origin of multiferroic spiral spin order in the RMnO3 perovskites. Phys. Rev. B.

[7] Van Aken, B. B., Palstra, T. T. M., Filippetti, A. & Spaldin, Na. The origin of ferroelectricity in magnetoelectric YMnO3. *Nat*. *Mater*. **3**, 164–170 (2004). 10.1038/nmat1080 14991018

[8] Coy LE (2016). Orientation dependent Ti diffusion in YNMO/STO thin films deposited by pulsed laser deposition. Appl. Surf. Sci..

[9] Coy LE (2015). Crystalline domains in epitaxial Y(Ni0.5Mn0.5)O3 thin films grown by PLD on different STO substrates. Appl. Surf. Sci..

[10] Langenberg E (2007). Thin films in ternary Bi–Mn–O system obtained by pulsed laser deposition. Mater. Sci. Eng. B.

[11] Langenberg E (2009). Epitaxial thin films of (Bi0.9La0.1)2NiMnO6 obtained by pulsed laser deposition. J. Magn. Magn. Mater..

[12] Booth RJ (2009). An investigation of structural, magnetic and dielectric properties of R2NiMnO6 (R=rare earth, Y). Mater. Res. Bull..

[13] Kitamura M (2009). Ferromagnetic properties of epitaxial La[sub 2]NiMnO[sub 6] thin films grown by pulsed laser deposition. Appl. Phys. Lett..

[14] Peña O, Barahona P, Gil V, Tartaj J, Moure C (2008). Magnetic behavior of solid solutions REMe0.50 Mn0.50 O3 (RE = Y, La, Pr, Nd, Eu, Gd, Er; Me = Ni,Co). Bol. la Soc. Esp. Ceram. y Vidr..

[15] Langenberg E (2010). Long-range order of Ni[sup 2 + ] and Mn[sup 4 + ] and ferromagnetism in multiferroic (Bi[sub 0.9]La[sub 0.1])[sub 2]NiMnO[sub 6] thin films. J. Appl. Phys..

[16] Gautam A, Singh K, Sen K, Kotnala RK, Singh M (2012). Magneto-electric properties of Nd substituted BiFeO3 polycrystalline samples. J. Alloys Compd..

[17] Schmidt R (2012). Magnetoimpedance spectroscopy of epitaxial multiferroic thin films. Phys. Rev. B.

[18] Langenberg E (2012). Dielectric properties of (Bi0.9La0.1)2NiMnO6 thin films: Determining the intrinsic electric and magnetoelectric response. Phys. Rev. B.

[19] Rao CNR (2012). Sundaresan, a. & Saha, R. Multiferroic and Magnetoelectric Oxides: The Emerging Scenario. J. Phys. Chem. Lett..

[20] Eerenstein W, Mathur ND, Scott JF (2006). Multiferroic and magnetoelectric materials. Nature.

[21] Coy LE (2016). Dielectric characterization of multiferroic magnetoelectric double-perovskite Y(Ni 0.5 Mn 0.5)O 3 thin films. Appl. Phys. Lett..

[22] Ahmed T (2017). Magnetic, electronic, and optical properties of double perovskite Bi 2 FeMnO 6. APL Mater..

[23] Bibes M, Barthélémy A (2008). Multiferroics: Towards a magnetoelectric memory. Nat. Mater..

[24] Spaldin NA, Cheong S-W, Ramesh R (2010). Multiferroics: Past, present, and future. Phys. Today.

[25] Nagarajan V (2003). Dynamics of ferroelastic domains in ferroelectric thin films. Nat. Mater..

[26] Baek SH (2010). Ferroelastic switching for nanoscale non-volatile magnetoelectric devices. Nat. Mater..

[27] Agar JC (2016). Highly mobile ferroelastic domain walls in compositionally graded ferroelectric thin films. Nat. Mater..

[28] Wang C (2016). Ferroelastic switching in a layered-perovskite thin film. Nat. Commun..

[29] Chen AT, Zhao YG (2016). Research Update: Electrical manipulation of magnetism through strain-mediated magnetoelectric coupling in multiferroic heterostructures. APL Mater..

[30] Zhu Y (2013). Research Updates: Epitaxial strain relaxation and associated interfacial reconstructions: The driving force for creating new structures with integrated functionality. APL Mater..

[31] Fernandes Vaz CA, Staub U (2013). Artificial multiferroic heterostructures. J. Mater. Chem. C.

[32] Sharma Y (2017). Long-range Stripe Nanodomains in Epitaxial (110) BiFeO3 Thin Films on (100) NdGaO3 Substrate. Sci. Rep..

[33] Everhardt AS, Matzen S, Domingo N, Catalan G, Noheda B (2016). Ferroelectric Domain Structures in Low-Strain BaTiO 3. Adv. Electron. Mater..

[34] Jia T, Kimura H, Cheng Z, Zhao H (2016). Switching of both local ferroelectric and magnetic domains in multiferroic Bi0.9La0.1FeO3 thin film by mechanical force. Sci. Rep..

[35] Liu M (2013). Non-volatile ferroelastic switching of the Verwey transition and resistivity of epitaxial Fe3O4/PMN-PT (011). Sci. Rep..

[36] Xu R (2014). Ferroelectric polarization reversal via successive ferroelastic transitions. Nat. Mater..

[37] Malgrange C, Glogarova M (1972). Study Of Ferroelectric Domains In Gadolinium Molybdate. Le J. Phys. Colloq..

[38] Dvořák V (1971). A Thermodynamic Theory of Gadolinium Molybdate. Phys. Status Solidi.

[39] Cross LE, Fouskova A, Cummins SE (1968). Gadolinium Molybdate, a New Type of Ferroelectric Crystal. Phys. Rev. Lett..

[40] Megumi K, Yumoto H, Ashida S, Akiyama S, Furuhata Y (1974). Phase equilibrium diagram for the system Gd2O3-MoO3. Mater. Res. Bull..

[41] Aizu K, Kumada A, Yumoto H, Ashida S (1969). Simultaneous Ferroelectricity and Ferroelasticity of Gd 2 (MoO 4) 3. J. Phys. Soc. Japan.

[42] Dudnikova VB, Zharikov EV (2017). Atomistic simulation of ferroelectric–ferroelastic gadolinium molybdate. Phys. Solid State.

[43] Kumar P, Gupta BK (2015). New insight into rare-earth doped gadolinium molybdate nanophosphor assisted broad spectral converters from UV to NIR for silicon solar cells. RSC Adv..

[44] Zhao X (2007). Luminescent properties of Eu3 + doped α-Gd2(MoO4)3 phosphor for white light emitting diodes. Opt. Mater. (Amst)..

[45] Honma T, Tsukada Y, Komatsu T (2010). Two-dimensional Raman imaging for periodic domain structures in laser-patterned ferroelastic β′-(Sm,Gd)2(MoO4)3 crystal lines in glass. Opt. Mater. (Amst)..

[46] Tsukada Y, Honma T, Komatsu T (2009). Self-organized periodic domain structure for second harmonic generations in ferroelastic β′-(Sm,Gd)2(MoO4)3 crystal lines on glass surfaces. Appl. Phys. Lett..

[47] Ko SW, Mourey DA, Clark T, Trolier-McKinstry S (2010). Synthesis, characterization, and dielectric properties of β-Gd2(MoO4)3 thin films prepared by chemical solution deposition. J. Sol-Gel Sci. Technol..

[48] Suzuki F, Honma T, Komatsu T (2014). Unique crystal growth with crystal axis rotation in multi-ferroic β′-(Sm,Gd)2(MoO4)3 narrow lines patterned by lasers in glass. J. Phys. Chem. Solids.

[49] Kappert EJ (2015). Formation and prevention of fractures in sol–gel-derived thin films. Soft Matter.

[50] Pan G (2017). Synthesis and thermochromic property studies on W doped VO2 films fabricated by sol-gel method. Sci. Rep..

[51] De Dobbelaere, C. *et al*. in *Nanoscale Ferroelectrics and Multiferroics* 163–199, doi:10.1002/9781118935743.ch7 (John Wiley & Sons, Ltd, 2016).

[52] Eason, R. *Pulsed Laser Deposition of Thin Films: Applications-Led Growth of Functional Materials*. *Pulsed Laser Deposition of Thin Films: Applications-Led Growth of Functional Materials* doi:10.1002/9780470052129 (John Wiley & Sons, Inc., 2006).

[53] Lorenz M, Ramachandra Rao MS (2014). 25 years of pulsed laser deposition. J. Phys. D. Appl. Phys..

[54] Haindl S, Hanzawa K, Sato H, Hiramatsu H, Hosono H (2016). *In-situ* growth of superconducting SmO1–x F x FeAs thin films by pulsed laser deposition. Sci. Rep..

[55] Zhang K (2016). Vertical La0.7Ca0.3MnO3 nanorods tailored by high magnetic field assisted pulsed laser deposition. Sci. Rep..

[56] Wang W (2015). Interfacial reaction control and its mechanism of AlN epitaxial films grown on Si(111) substrates by pulsed laser deposition. Sci. Rep..

[57] Jin T (2017). Real-Time and Label-Free Chemical Sensor-on-a-chip using Monolithic Si-on-BaTiO3 Mid-Infrared waveguides. Sci. Rep..

[58] Ventura J (2015). Heterogeneous distribution of B-site cations in BaZrxTi1−xO3 epitaxial thin films grown on (001) SrTiO3 by pulsed laser deposition. Appl. Surf. Sci.

[59] Voevodin, A. A., Zabinski, J. S., Jones, J. G. & Norton, D. P. *Pulsed Laser Deposition of Thin Films*. *Pulsed Laser Deposition of Thin Films: Applications-Led Growth of Functional Materials*, doi:10.1002/0470052120 (John Wiley & Sons, Inc., 2006).

[60] Sánchez F, Ocal C, Fontcuberta J (2014). Tailored surfaces of perovskite oxide substrates for conducted growth of thin films. Chem. Soc. Rev..

[61] Wisniewski W, Seidel S, Patzig C, Rüssel C (2017). Surface Crystallization of a MgO/Y2O3/SiO2/Al2O3/ZrO2 Glass: Growth of an Oriented β-Y2Si2O7 Layer and Epitaxial ZrO2. Sci. Rep..

[62] Enriquez E (2017). Oxygen Vacancy-Tuned Physical Properties in Perovskite Thin Films with Multiple B-site Valance States. Sci. Rep..

[63] Hensling FVE, Xu C, Gunkel F, Dittmann R (2017). Unraveling the enhanced Oxygen Vacancy Formation in Complex Oxides during Annealing and Growth. Sci. Rep..

[64] Mitchell DRG (2008). Circular Hough transform diffraction analysis: A software tool for automated measurement of selected area electron diffraction patterns within Digital Micrograph^TM^. Ultramicroscopy.

[65] Green AE (2017). Growth Mechanism of Dendritic Hematite via Hydrolysis of Ferricyanide. Cryst. Growth Des..

[66] Huan TN (2017). A Dendritic Nanostructured Copper Oxide Electrocatalyst for the Oxygen Evolution Reaction. Angew. Chemie.

[67] Van Loo W (1975). Crystal growth and electrical conduction of PbMoO4 and PbWO4. J. Solid State Chem..

[68] Zeng HC, Chong TC, Lim LC, Kumagai H, Hirano M (1994). Pseudo-dendritic growth in lead molybdate single crystal by Czochralski technique. J. Cryst. Growth.

[69] Wunderlich W, Foitzik AH, Heuer AH (1993). On the quantitative EDS analysis of low carbon concentrations in analytical TEM. Ultramicroscopy.

[70] Vincent Crist B (1999). Handbooks of Monochromatic XPS Spectra Volume 1 - The Elements and Native Oxides. Handb. Elem. Nativ. Oxides.

[71] Denev SA, Lummen TTA, Barnes E, Kumar A, Gopalan V (2011). Probing Ferroelectrics Using Optical Second Harmonic Generation. J. Am. Ceram. Soc..

[72] Rubano A (2015). Polar asymmetry of La (1− δ) Al (1 + δ) O 3/SrTiO 3 heterostructures probed by optical second harmonic generation. Appl. Phys. Lett..

[73] Jones R, Adams JM, Evans S (1987). A new barium molybdate phase. Mater. Res. Bull..

[74] Nishioka H (1997). Femtosecond continuously tunable second harmonic generation over the entire-visible range in orthorhombic acentric Gd2(MoO4)3 crystals. Appl. Phys. Lett..

