# Supplementary material for: Second Harmonic Generation Response in Thermally reconstructed Multiferroic β′- Gd2(MoO4)3 Thin Films
Source: Sci Rep. 2017 Sep 18;7:11800. doi: 10.1038/s41598-017-12370-y (PMC5603551; doi:10.1038/s41598-017-12370-y)
Supplement: Supplementary file 1 — Supplementary information [file 41598_2017_12370_MOESM1_ESM.pdf]

Second Harmonic Generation Response in Thermally reconstructed Multiferroic β′-$Gd_2(MoO_3)_4$ Thin Films

Emerson Coy[1], Piotr Graczyk[1,2], Luis Yate[3], Karol Załęski[1], Jacek Gapiński[1,2], Piotr Kuświk[4], Sławomir Mielcarek[2], Feliks Stobiecki[4], Bogusław Mróz[1,2], Cesar Ferrater[5], Stefan Jurga[1,6]*

1 *NanoBioMedical Centre, Adam Mickiewicz University, Umultowska 85, 61-614 Poznań, Poland.*
2 *Faculty of Physics, Adam Mickiewicz University, Umultowska 85, 61-614 Poznań, Poland.*
3 *CIC biomaGUNE, Paseo Miramón 182, 20009, San Sebastián, Spain.*
4 *Institute of Molecular Physics, Polish Academy of Sciences, M. Smoluchowskiego 17, 60-179 Poznań, Poland.*
5 *Departament de Física Aplicada i Òptica, Universitat de Barcelona, Martí i Franquès 1, Barcelona, Spain.*
6 *Department of Macromolecular Physics, Faculty of Physics, Adam Mickiewicz University, Umultowska 85, 61-614 Poznań, Poland*

Corresponding Author *: coyeme@amu.edu.pl

Key Words: Ferroelastic; Ferroelectric; Molybdates; Gadolinium; Surface reconstruction;

Table of Contents:

Figure S1 -	GIXRD plots of samples deposited at different temperatures	page S-2
Figure S2 -	Atomic Force Micrographs	page S-3
Figure S3-	AFM – Thickness dependent	page S-4
Figure S4 -	TEM - EDX mappings	page S-5
Table S1 -	XPS atomic composition	page S-6

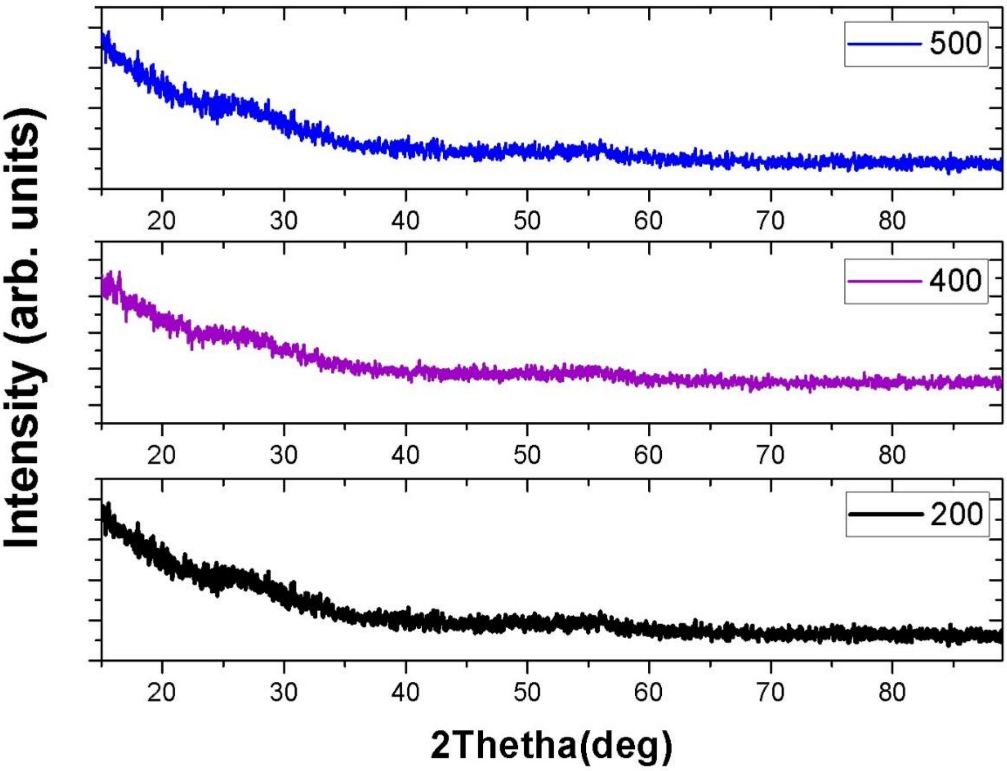

Figure S1: GI-XRD patterns for samples deposited by PLD at different temperatures.

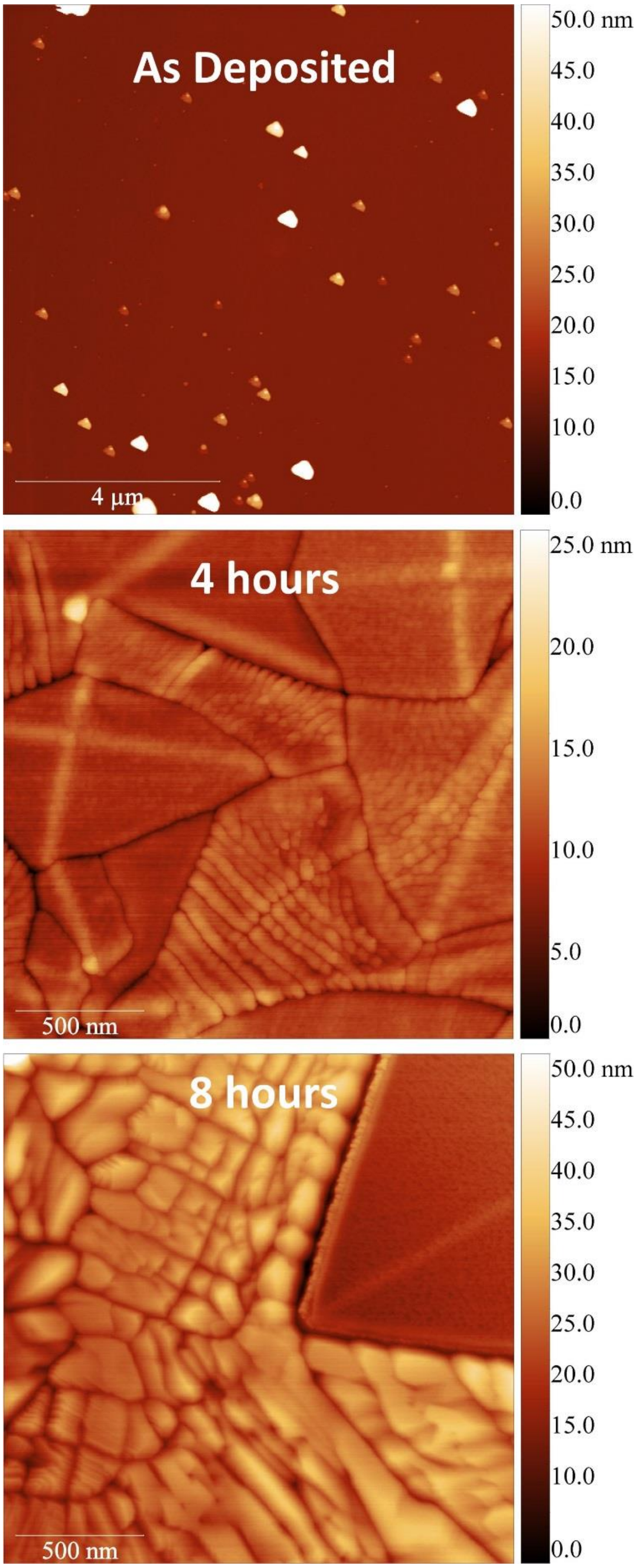

Figure S2: Atomic Force Micrographs of as deposited surface (top), 4 hours (middle) and 8 hours (bottom)

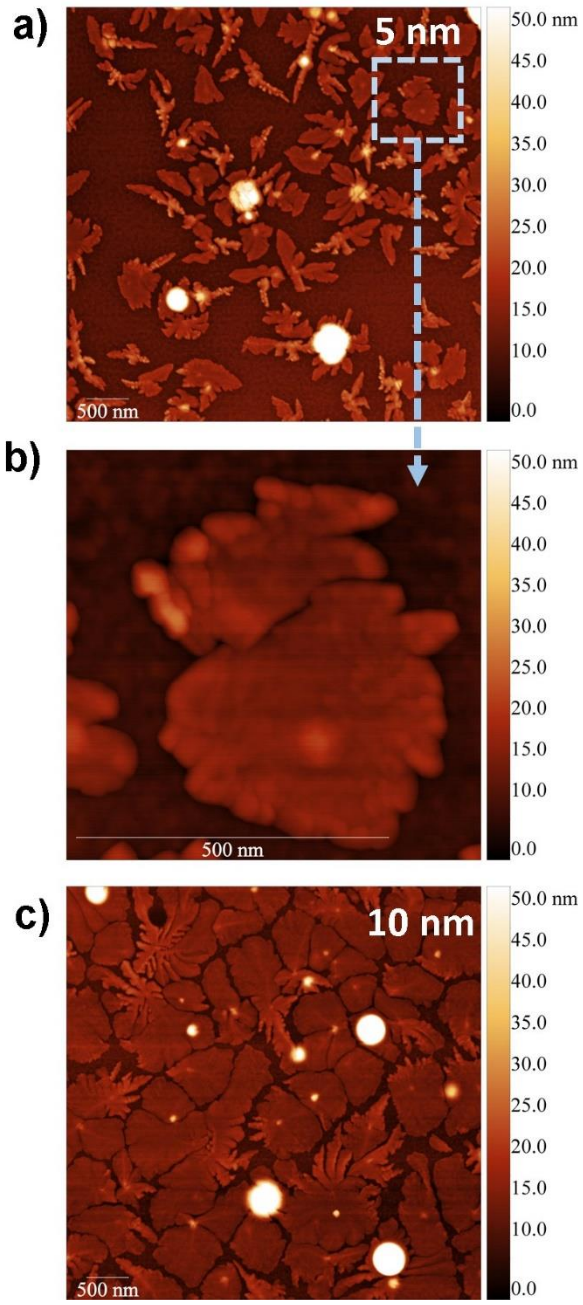

Figure S3: Atomic Force Micrographs samples with different thickness. a),b) 5nm and c) 10 nm. Samples were annealed for 2 hours.

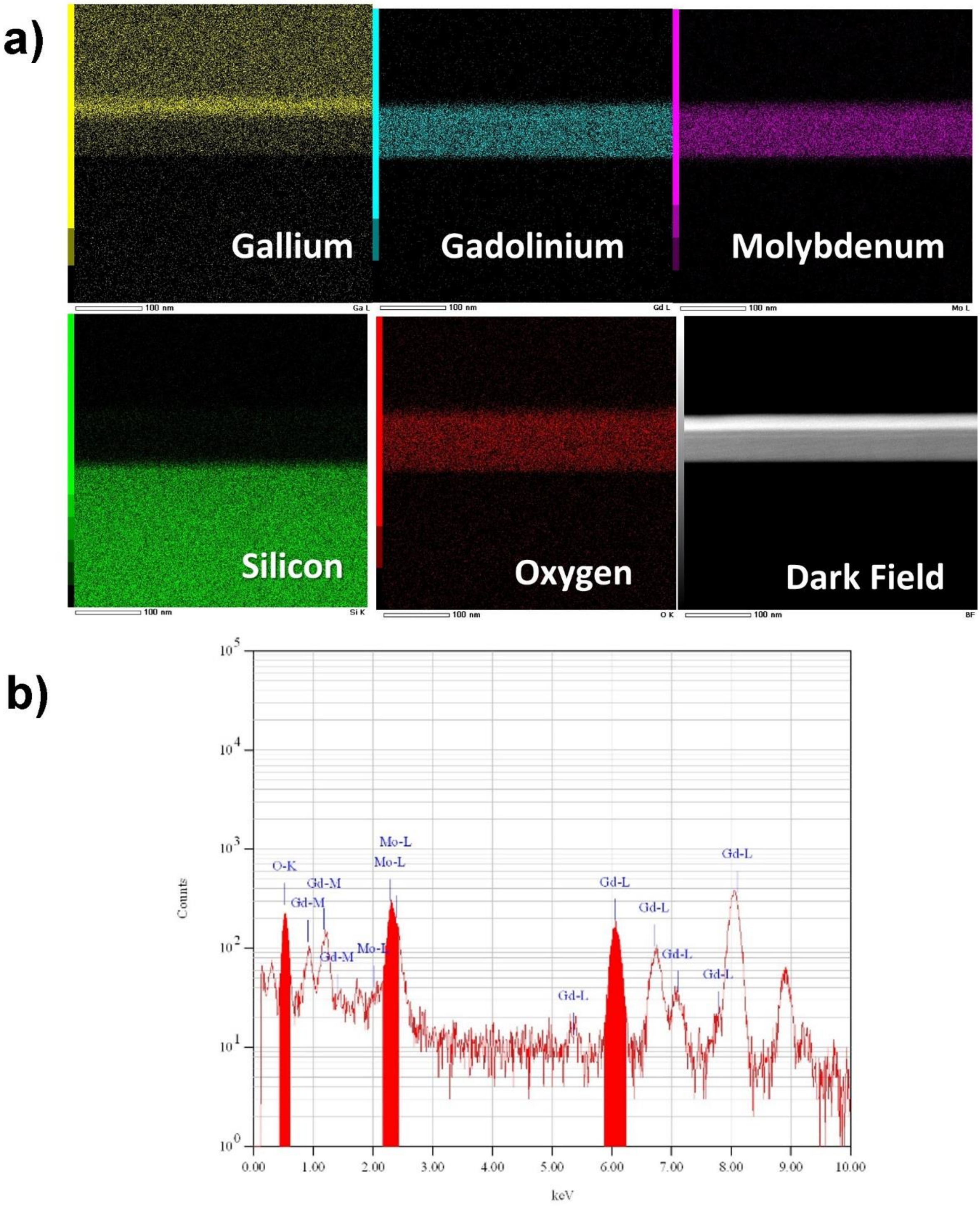

Figure S4: a) TEM - EDX mappings filtered by specific element b) total spectra, un labeled peaks ≈9 keV are Cu grid.

Table S1 - XPS atomic composition: The normalized chemical compositions from Me-O component of the O 1s ignoring C=O components.

Sample	Gd (at.%)	Mo (at.%)	O (at.%)
**$Gd_2(MoO_3)_4$**	11,1	22,2	66,7
**2h**	9,5	6,4	84,1
**4h**	9,4	11,4	79,2
**8h**	14,9	22,4	62,8
